# Single-cell transcriptomics reveals the heterogeneity and function of mast cells in human ccRCC

**DOI:** 10.3389/fimmu.2024.1494025

**Published:** 2025-01-07

**Authors:** Xiyu Song, Jianhua Jiao, Jiayang Qin, Wei Zhang, Weijun Qin, Shuaijun Ma

**Affiliations:** ^1^ Department of Urology, Xijing Hospital, Fourth Military Medical University, Xi’an, Shaanxi, China; ^2^ National Translational Science Center for Molecular Medicine & Department of Cell Biology, Fourth Military Medical University, Xi’an, Shaanxi, China; ^3^ Xijing Innovation Research Institute, Fourth Military Medical University, Xi’an, Shaanxi, China; ^4^ West China School of Medicine, West China Hospital, Sichuan University, Chengdu, China

**Keywords:** mast cell, clear cell renal cell carcinoma, single-cell RNA sequencing, heterogeneity, spatial transcriptomics, prognosis

## Abstract

**Introduction:**

The role of mast cells (MCs) in clear cell renal carcinoma (ccRCC) is unclear, and comprehensive single-cell studies of ccRCC MCs have not yet been performed.

**Methods:**

To investigate the heterogeneity and effects of MCs in ccRCC, we studied single-cell transcriptomes from four ccRCC patients, integrating both single-cell sequencing and bulk tissue sequencing data from online sequencing databases, followed by validation via spatial transcriptomics and multiplex immunohistochemistry (mIHC).

**Results:**

We identified four MC signature genes (TPSB2, TPSAB1, CPA3, and HPGDS). MC density was significantly greater in ccRCC tissues than in normal tissues, but MC activation characteristics were not significantly different between ccRCC and normal tissues. Activated and resting MCs were defined as having high and low expression of MC receptors and mediators, respectively, whereas proliferating MCs had high expression of proliferation-related genes. The overall percentage of activated MCs in ccRCC tissues did not change significantly but shifted toward a more activated subpopulation (VEGFA^+^ MCs), with a concomitant decrease in proliferative MCs (TNF^+^ MCs) and resting MCs. An analysis of the ratio of TNF^+^/VEGFA^+^ MCs in tumors revealed that MCs exerted antitumor effects on ccRCC. However, VEGFA^+^MC was produced in large quantities in ccRCC tissues and promoted tumor angiogenesis compared with adjacent normal tissues, which aroused our concern. In addition, MC signature genes were associated with a better prognosis in the KIRC patient cohort in the TCGA database, which is consistent with our findings. Furthermore, the highest level of IL1B expression was observed in macrophages in ccRCC samples, and spatial transcriptome analysis revealed the colocalization of VEGFA^+^ MCs with IL1B^+^ macrophages at the tumor–normal interface.

**Discussion:**

In conclusion, this study revealed increased MC density in ccRCC. Although the proportion of activated MCs was not significantly altered in ccRCC tissues compared with normal tissues, this finding highlights a shift in the MC phenotype from CTSG^high^MCs to more activated VEGFA+MCs, providing a potential therapeutic target for inhibiting ccRCC progression.

## Introduction

Renal cell carcinoma (RCC) is one of the ten most common malignant tumors worldwide, with clear cell renal cell carcinoma (ccRCC) accounting for approximately 75% of RCC cases and a majority of kidney cancer deaths (1). ccRCC is a type of cancer with severe immune cell infiltration (2). Immune checkpoint blockade (ICB) therapy, an adaptive immunity approach, can effectively improve patient survival, highlighting the importance of the ccRCC immune microenvironment (3). Despite advances in current immunotherapies, their efficacy in treating ccRCC remains limited. To address this challenge, it is critical to obtain a deeper understanding of the interactions between various cells and molecules in the ccRCC tumor microenvironment (TME) and to develop new targets for immunotherapy.

Mast cells (MCs) are histochemically distinctive tissue-resident effector cells originating from the hematopoietic system (4). MCs serve as an ancient component of the immune system, as MC-like cells exist hundreds of millions of years before adaptive immunity (5). MCs are known to be the major effector cells involved in IgE-driven type 1 hypersensitivity reactions and play crucial roles in allergic diseases (6). In addition, MCs are associated with the development of inflammation, wound healing, host defense, vasodilatory tone regulation, autoimmune diseases, and cancer (7). However, the role of MCs in cancers remains controversial. There are few studies on MCs in ccRCC, and no consensus conclusions have been reached (8, 9). Therefore, further investigations are necessary to obtain a more comprehensive understanding of the effects of MCs in ccRCC.

The growth of single-cell RNA sequencing (scRNA-seq) has provided a detailed understanding of the phenotypic and functional heterogeneity of single cells and has revolutionized our understanding of the immune landscape of tumors (10). In the context of ccRCC, scRNA-seq has been widely used to study the phenotypic and functional characteristics of tumor cells, stromal cells (endothelial and fibroblasts, etc.), and immune cells (endothelial and fibroblasts, etc.) in the TME (11–13). However, the phenotypic and functional heterogeneity of MCs in the TME of ccRCC remains unexplored.

Our study aimed to fill this gap by utilizing scRNA-seq technologies to recognize the heterogeneity of MCs in the ccRCC TME. Our findings represent the first specific articulation of antitumor and protumor signals of MCs in ccRCC, revealing the underlying mechanisms of this dynamic balance and the protective role of MCs in patient prognosis. Furthermore, we identified the geographic regions of each MC subset in ccRCC for the first time via spatial transcriptomics and validated our findings in 15 ccRCC patients via multiplex immunohistochemistry (mIHC). Our study offers valuable novel insights into the complex interactions in the immune system of ccRCC patients and has important implications for the development of new therapeutic approaches for ccRCC.

## Identification of MC marker genes and increased MC density in ccRCC

**Figure 1:**
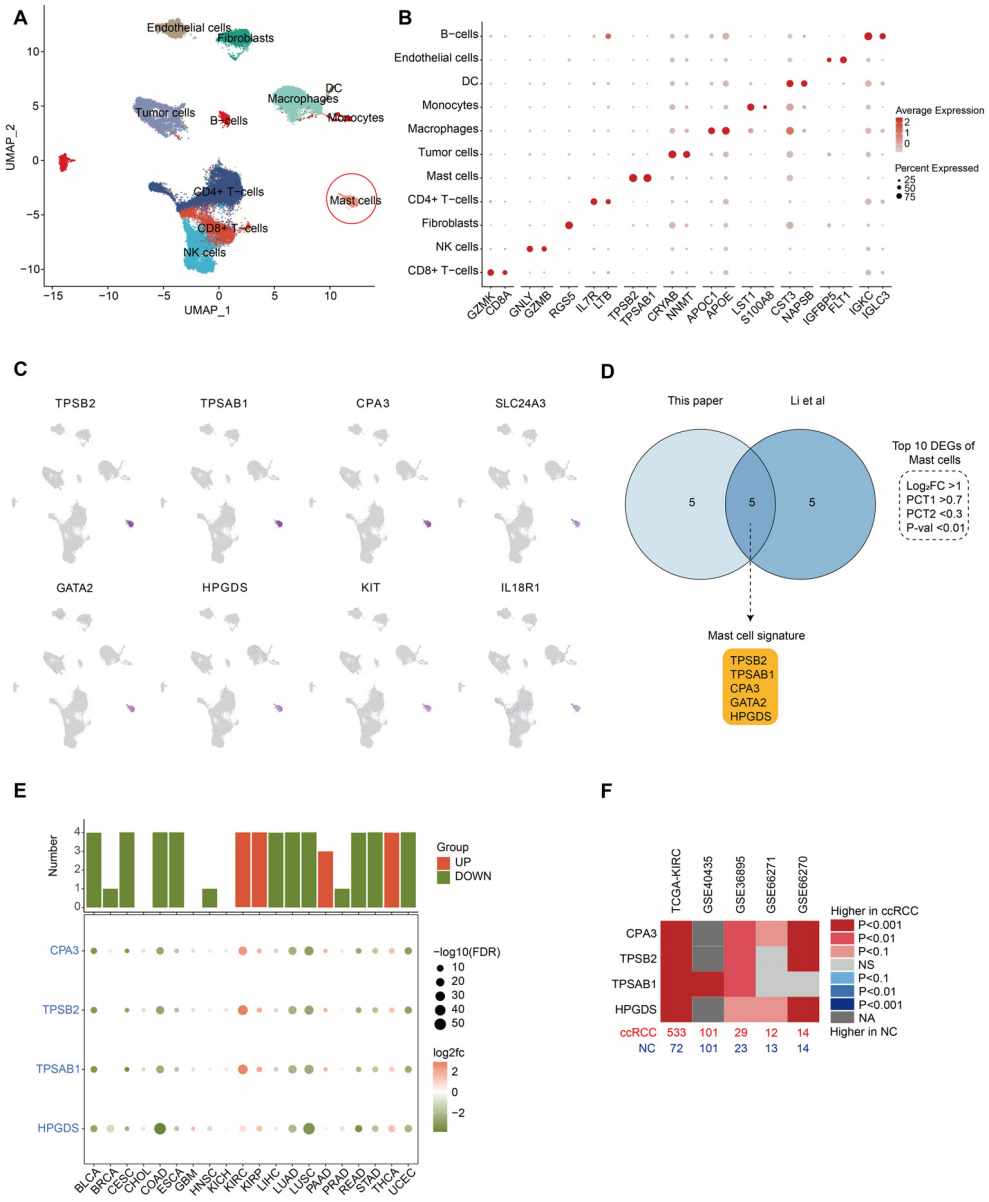
Identification of MC marker genes and increased MC density in ccRCC. **(A)** UMAP plot showing the major cell types identified in our study. The red circles indicate MCs. **(B)** Dot plots displaying marker genes of each major cell type in our study. All the marker genes are listed in **Supplementary Table S2**. **(C)** UMAP plots showing marker gene expression for MCs. **(D)** Venn diagram of differentially expressed genes (DEGs) of MCs in our study and the study by Li et al. The intersection indicates the five MC marker genes, and the criteria for filtering DEGs are depicted in the dashed line. **(E)** Dot plots showing the expression of MC marker genes in each cancer (tumor vs. normal). Histograms show the number of statistically significant genes. Red histograms indicate increased tumor expression, and green histograms indicate decreased tumor expression (p values < 0.05). **(F)** Heatmap showing the expression of MC marker genes in 5 bulk RNA-seq cohorts of ccRCC patients (tumor vs. normal). Red represents an increase in tumor expression, sample size (bottom), and dataset source (top).

In this study, we collected tumor (tumor core and tumor rim) and adjacent normal tissues from four ccRCC patients for single-cell RNA sequencing (scRNA-seq) (**Supplementary Table S1**). To improve the resolution of immune cells, we prepared scRNA-seq libraries by mixing CD45^+^ immune cells and CD45^-^ cells nine-to-one for each sample. After stringent quality control, the transcriptomes of 64,596 cells were captured, and 1,975 MCs were identified, representing 3.6% of the total number of cells. Data integration revealed major clusters on the basis of marker genes, including CD8^+^ T cells (CD8A, GZMK), CD4^+^ T cells (IL7R, LTB), natural killer (NK) cells (GZMB, GNLY), B cells (IGKC, IGLC3), MCs (TPSAB1, TPSB2), macrophages (APOC1, APOE), monocytes (LST1, S100A8), DCs (CST3, NAPSB), endothelial cells (IGFBP5, FLT1), fibroblasts (RGS5) and tumor cells (CRYAB, NNMT) (**Figures 1A, B**; **Supplementary Table S2**). Each cluster contained cells from all of the patients, indicating that there were no significant patient-specific batch effects (**Supplementary Figures S1A-S1C**).

To identify MC signature genes, we established rigorous differential gene screening criteria (P value < 0.01, log2-fold change (log2FC) > 1, PCT1 > 0.7, and PCT2 < 0.3). In our single-cell dataset, eight of the top 10 differentially expressed genes (DEGs) of MCs matched (**Figure 1C**; **Supplementary Table S2**). Further comparison with a recent ccRCC study by Li et al. in an online sequencing database revealed that five genes (TPSAB1, TPSB2, CPA3, HPGDS, and GATA2) were common DEGs (**Figure 1D**). The clinical data of the patients in both single-cell datasets are shown in **Supplementary Table S1**. To define MCs accurately, we reviewed previous reports of these genes (14–16). Among these, GATA2 is expressed in various cell types, including hematopoietic stem cells (HSCs), myeloid cells, hepatocytes and neurons, and is not a specific marker for MCs (17, 18). GATA2 was identified as a DEG in our cell cluster and is highly likely to be used as a marker for HSCs, as MCs originate from HSCs in the bone marrow. Therefore, we ultimately discarded GATA2 and defined TPSAB1, TPSB2, CPA3 and HPGDS as MC signature genes. The signature genes can also be confirmed in 3 cohorts (GSE210038, GSE202374, and GSE207493) (**Supplementary Figure S2**).

The MC signature genes were regarded as MC markers to evaluate the MC density in bulk RNA-seq samples from tumor and normal tissues. All four MC signature genes that reached statistical significance (P < 0.05) were considered plausible. The results indicated that MC signature genes were decreased in most tumors and were significantly increased only in KIRC, KIRP, and THCA (abbreviations of the cancer are shown in **Supplementary Table S3**), with the most significant increase in KIRC (**Figure 1E**). Follow-up analyses in other KIRC cohorts confirmed the significant increase in MC density in ccRCC patients (**Figure 1F**).

In conclusion, we identified four reliable MC signature genes as MC markers, and our findings consistently indicate an increase in MC density in ccRCC. Notably, the manifestation of MCs in ccRCC is at odds with that in numerous other systemic tumors, which deserves more in-depth exploration.

## MC activation in ccRCC

**Figure 2:**
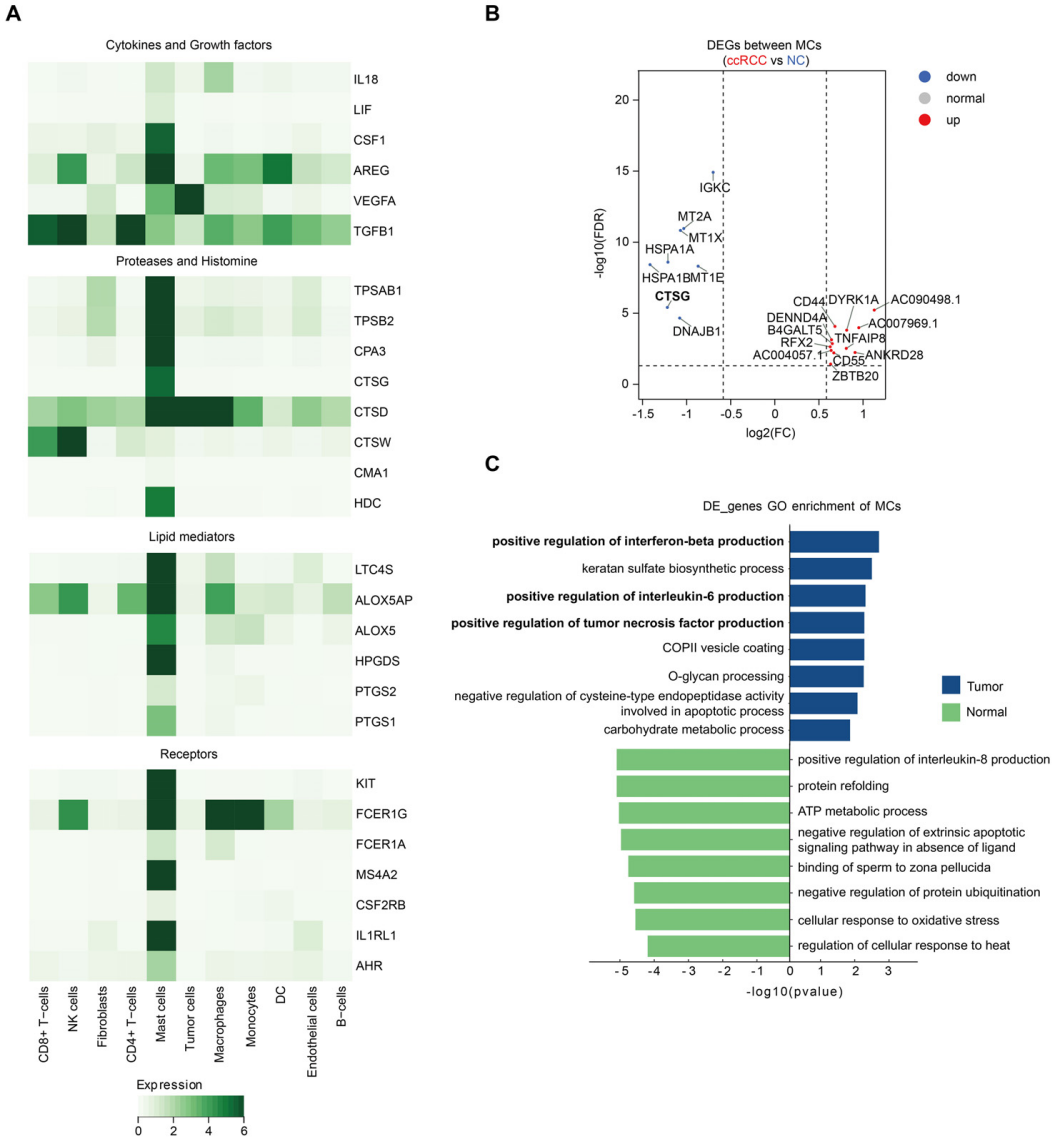
MC activation in ccRCC. **(A)** Heatmap displaying the expression of MC-related receptor and mediator genes across different major cell types. **(B)** Volcano plot showing the DEGs between ccRCC MCs and NC MCs from our study. All the genes are listed in **Supplementary Table S4**. **(C)** Enriched pathways in ccRCC MCs and NC MCs according to the GO pathway enrichment analysis.

To better understand the activation characteristics of MCs during ccRCC progression, we analyzed the expression of MC-related receptor and mediator genes (6, 19). The results revealed that MCs were the major cell population expressing CSF2RB and AHR, with the highest expression levels of IL-33 (IL1RL1) and the KIT receptor. Notably, MCs are also a unique cell population expressing all subunits of the high-affinity IgE receptor FcϵR1 (i.e., MS4A2, FCER1A, and FCER1G). In addition, MCs highly expressed characteristic proteases, including TPSAB1, TPSB2, CPA3, CTSG, CTSD, CTSW, and CMA1, with CTSD and CTSW also being enriched in other cell populations. MCs also presented high expression of genes related to histamine biosynthesis (HDC), leukotriene biosynthesis (ALOX5, ALOX5OP, and LTC4S), and prostaglandin biosynthesis (PTGS1, PTGS2 and HPGDS). Moreover, MCs highly expressed multiple chemokines, cytokines, and growth factors (including IL18, LIF, CSF1, AREG, VEGFA, and TGFB1). Among them, MCs were the only cell population encoding LIF and CSF1 mRNAs (**Figure 2A**).

To understand more deeply the changes in MCs during ccRCC tumorigenesis, we compared the gene expression differences between MCs in ccRCC tissues and adjacent normal tissues via single-cell data. Genes that met the criteria of a P value < 0.05 and log 2FC> 0.25 were defined as DEGs. Among MCs from four ccRCC patients, the number of DEGs enriched in MCs from ccRCC patients was significantly greater than that enriched in normal tissues, suggesting that the expression of a wide range of genes in MCs increases during ccRCC progression (**Supplementary Table S4**). However, the expression of most MC receptor and mediator genes did not differ significantly between MCs in ccRCC tissues and normal tissues. The exception is CTSG, which encodes a histone protease and is significantly more highly expressed in adjacent normal tissues (**Figure 2B**).

To investigate the functional changes in MCs during progression from normal tissues to ccRCC, we performed enrichment analyses of DEGs. GO analysis revealed that pathways related to the positive regulation of interferon-beta, interleukin-6 and tumor necrosis factor (TNF) production were enriched in MCs in ccRCC (**Figure 2C**). Notably, previous reports have illustrated the importance of TNF^+^ mast cells in antitumor immunity (9). In addition, IL-6 promotes tumor growth, angiogenesis, and metastasis and reduces antitumor immune responses (20), whereas IFNβ has potent anticancer potential by inhibiting cell proliferation; controlling angiogenesis; and regulating apoptosis, differentiation, migration, and cell surface antigen expression (21). These findings remind us to evaluate the complexity of MCs in ccRCC.

Taken together, these findings suggest that MCs are activated to a comparable extent in tumor and adjacent normal tissues in ccRCC. The coexistence of MC-induced antitumor and protumor signals in the tumor immune microenvironment (TME) determines the complexity of the effects of tumor-associated MCs that ultimately influence tumor growth.

## Heterogeneity of MCs in ccRCC

**Figure 3:**
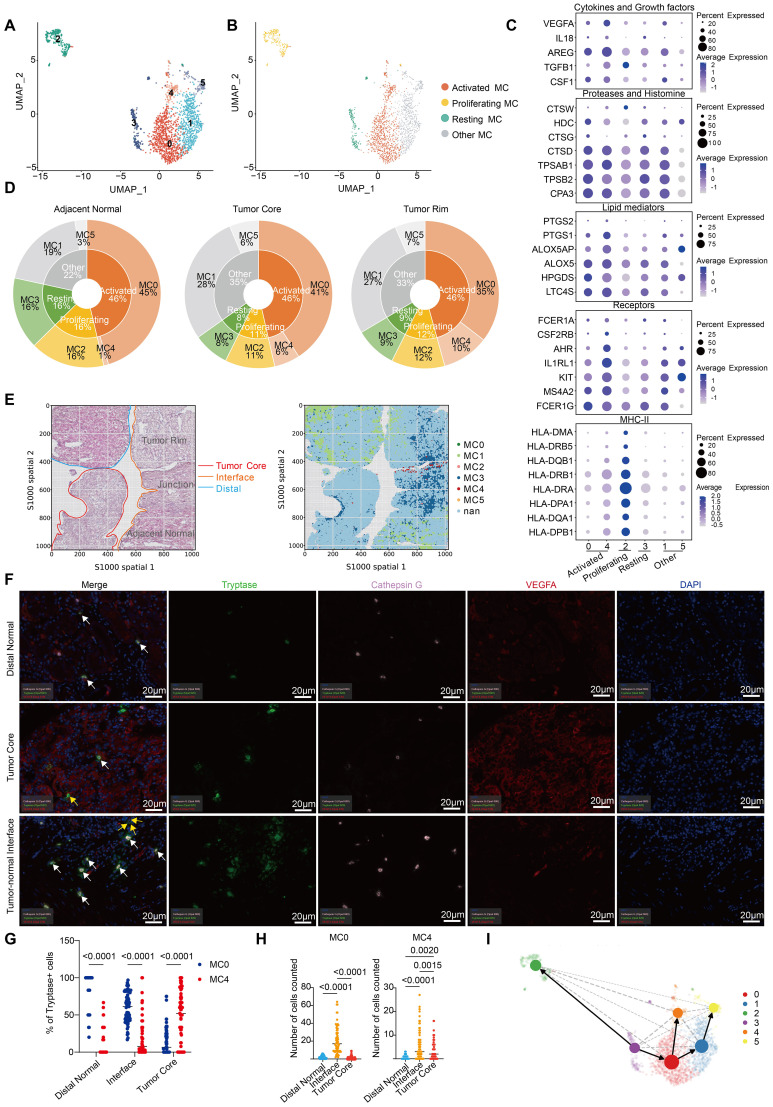
Heterogeneity of MCs in ccRCC. **(A)** UMAP plot of MCs colored by cluster in our study. **(B)** UMAP plot of MCs colored according to 4 distinct MC subtypes in our study. **(C)** Dot plot showing the expression of MC-related receptor and mediator genes across different MC clusters. **(D)** Pie charts showing the proportions of MC subtypes and clusters in different tissues. **(E)** Spatial plots showing HE staining (left) and the spatial distribution of each MC cluster (right). **(F)** Tissues from the distal normal kidney, tumor core and tumor-normal interface of 15 ccRCC patients were stained and analyzed by multiplex immunofluorescence (mIHC) for cathepsin G, tryptase and VEGFA. The white arrows point to cells that are positive for cathepsin G and tryptase, and the yellow arrows point to cells that are positive for VEGFA and tryptase. **(G)** Quantification of MC0 and MC4 cells (percentages of Cathepsin G+Tryptase+ or VEGFA+Tryptase+ cells pregated on Tryptase+ cells) by mIHC in different tissues. Each dot represents the average of two similar values, with an average of 5 dots per patient. **(H)** Absolute quantity of MC0 and MC4 cells (number of cathepsin G+Tryptase+ or VEGFA+Tryptase+ cells) determined via mIHC in different tissues. Each dot represents the average of two similar values, with an average of 5 dots per patient. The results were tested via multiple testing corrections. **(I)** UMAP with superimposed RNA velocity analysis of the MC subsets. The size of the dots represents the quantity of cells in each subset.

Single-cell analysis allowed us to characterize the phenotypes and functions of MCs during ccRCC in terms of MC heterogeneity. In our single-cell data, cluster analysis of 1975 MCs revealed 6 clusters, which corresponded to 4 different MC subtypes (**Figures 3A, B**; **Supplementary Table S2**). MC2 enriched for proliferation-related genes such as MKI67, CDK1, PCNA and TOP2A was named “proliferating MC” (**Supplementary Figure S3A**). MC0 and MC4, which mostly maintain the overall expression levels of MC signature proteases, lipids, receptors and cytokines, were named “activated MCs”, whereas MC3 was named “resting MCs” (**Figure 3C**; **Supplementary Table S5**). The mitochondrial gene (MT)-rich MC1 and MC5 strains were categorized as “other MCs” (**Supplementary Figure S3B**). Proliferating MCs (MC2) are also considered T-cell doublets because of their high expression of CD3D along with low expression of MC receptor and mediator genes (KIT, CPA3, AREG, IL1RL1, and MS4A2). The enrichment of MHCII-related genes (HLA-DRA, HLA-DPA1 and HLA-DPB1) in resting MCs (MC3) indicated a stronger antigen-presenting function (**Supplementary Figure S3C**) (22). In addition, chemokines such as CCL3, CCL4, CCL5 and CCL4L2 were significantly overexpressed in proliferating MCs and resting MCs, which may recruit CD8^+^ and CD4^+^ T cells into the tumor to perform antitumor functions (**Supplementary Figure S3C**). MC0 specifically highly expressed the histone CTSG, which has been previously reported in eosinophilic esophagitis (23). Notably, in activated MCs, MC4 cells presented greater expression levels of MC receptor and mediator genes than MC0 cells did (**Figure 3C**; **Supplementary Figure S3B**). The heterogeneity of MCs in ccRCC was also confirmed in 3 cohorts (GSE210038, GSE202374, and GSE207493) (**Supplementary Figures S3D, S3E**; **Supplementary Table S6**).

By comparing the distributions of various MC clusters in different tissues, we found that the proportion of activated MCs among the total MCs remained almost unchanged, which is consistent with the conclusion that MCs are activated to a comparable extent in tumor and adjacent normal tissues in ccRCC (**Figures 2B**; **3D**). Nevertheless, there was a transition from MC0 to MC4 in the tumor tissues of ccRCC patients. Specifically, high CTSG-expressing MC0 cells were mostly distributed in adjacent normal tissues, explaining the significantly increased expression of CTSG in normal tissues. In addition, the proportion of proliferating and resting MCs was significantly greater in adjacent normal tissues, whereas MC4 was almost absent (**Figure 3D**). This distribution pattern can also be observed in spatial transcriptomics data (the sample correspondence between the scRNA-seq data and spatial transcriptome data is shown in **Supplementary Figure S3F**). After mapping the spatial distribution of MCs in ccRCC tissues, on the basis of transcriptome expression and HE tissue information, we found that MC4 was mostly distributed at the tumor–normal interface (**Figure 3E**). To further determine the localization of activated MCs (MC0 and MC4), we performed mIHC analysis of tissue sections from 15 ccRCC patients and determined both the percentages and absolute numbers (**Figures 3F–H**; **Supplementary Figure S3G**; **Table 1**; **Supplementary Table S7**). Similarly, MC0 (Cathepsin G^+^Tryptase^+^) decreased from distal normal tissues to the tumor-normal interface to the tumor core, whereas the reverse was true for MC4 (VEGFA^+^Tryptase^+^) (**Figure 3G**). However, with respect to absolute quantity, we found that MC0 and MC4 tended to accumulate at the tumor-normal interface, which may be related to the invasion and metastasis of ccRCC (**Figure 3F**; **Supplementary Figures S3G, S3H**). Notably, these results have been shown to be independent of clinical characteristics such as patient age, sex, and disease stage (**Supplementary Table S8**).

**Table 1:** The strategies of mIHC.

Order	Antibody	Company	Product code	Dilution	Incubation	TSA
1	Cathepsin G	Abcam	ab282105	1:5000	60 min, 37°C	690
2	VEGFA	Proteintech	19003-1-AP	1:500	60 min, 37°C	570
3	Tryptase	HUABio	ET1610-64	1:4000	60 min, 37°C	620
4	DAPI	Phenoptics	NEL811001KT	2 drops/ml	10 min, room temperature	—

Order	Antibody	Company	Product code	Dilution	Incubation	TSA
1	CD68	Abcam	ab213363	1:1000	60 min, 37°C	690
2	VEGFA	Proteintech	19003-1-AP	1:500	60 min, 37°C	650
3	Il-1 beta	HUABio	ET1701-39	1:200	60 min, 37°C	570
4	Tryptase	HUABio	ET1610-64	1:4000	60 min, 37°C	620
5	DAPI	Phenoptics	NEL811001KT	2 drops/ml	10 min, room temperature	—

Furthermore, via RNA velocity analysis (24), we confirmed the transition from resting MCs to proliferating and activated MCs, as well as from MC0 MCs to MC4 MCs in activated MCs (**Figure 3I**). In conclusion, we revealed the heterogeneity of MCs during ccRCC. Our results demonstrate that there is no significant causal relationship with the clinical characteristics of ccRCC patients, but whether a similar pattern exists in other types of tumors needs to be further explored.

## A high MC signature is associated with favorable outcomes in ccRCC patients

**Figure 4:**
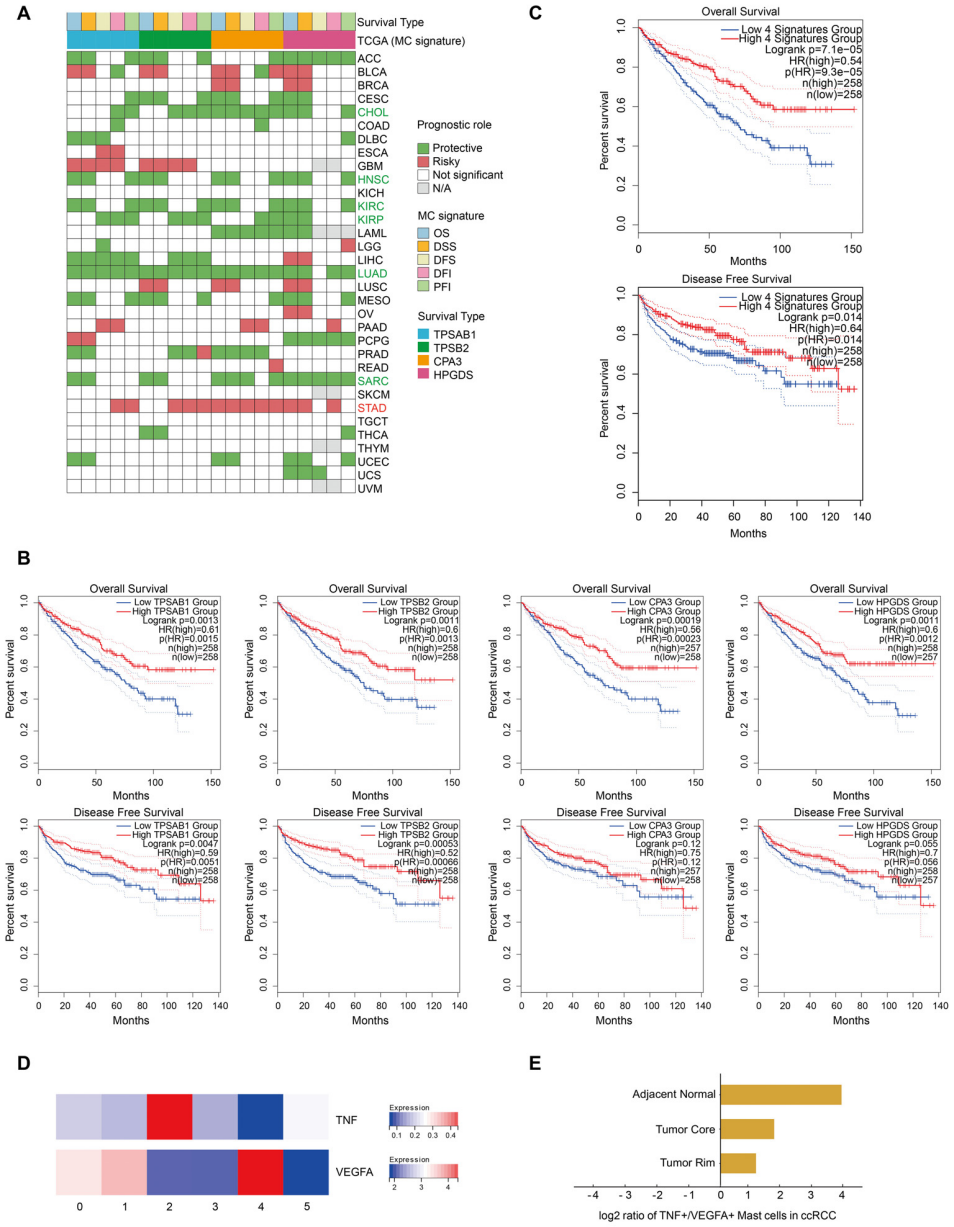
A high MC signature is associated with favorable outcomes in ccRCC patients. **(A)** Heatmap showing the correlation between the expression of each MC signature gene and overall survival (OS), disease-specific survival (DSS), disease-free survival (DFS), disease-free interval (DFI), and progression-free interval (PFI) on the basis of Kaplan‒Meier models and univariate Cox regression. Green indicates protective factors, while red represents risk factors for patient prognosis. p values < 0.05. **(B)** Kaplan‒Meier overall survival and disease-free survival curves grouped by each MC signature gene (TPSAB1, TPSB2, CPA3 and HPGDS) in KIRC. **(C)** Kaplan‒Meier overall survival and disease-free survival curves of the MC signature in our study. **(D)** Heatmap displaying the expression of proliferation-related genes across different MC clusters. **(E)** Bar plot showing the ratios of TNF+ MCs to VEGFA+ MCs in different tissues.

The role of MCs in cancer prognosis remains controversial, as they can promote or inhibit tumor growth in different TMEs (25). On the basis of the Kaplan−Meier analysis of the TCGA pancancer data, we used univariate Cox regression to assess the effect of the MC signature (four marker genes) on the prognosis of different cancers in terms of disease-specific survival (DSS), overall survival (OS), disease-free interval (DFI), disease-free survival (DFS), and progression-free interval (PFI). The results were considered reliable if at least half of the indicators reached statistical significance (p < 0.05). The results indicated that the MC signature had a significant protective effect on six cancer types, including CHOL, HNSC, KIRC, KIRP, LUAD, and SARC, whereas it was associated with a poor prognosis in only patients with STAD (**Figure 4A**). Furthermore, Kaplan−Meier analysis of TCGA-KIRC data revealed that a high MC signature and high expression of each of the four MC marker genes were associated with better overall and disease-free survival in the KIRC cohort (**Figures 4B, C**).

More importantly, previous studies have emphasized the importance of the ratio of TNF^+ MCs^ to VEGFA^+^ MCs for their cancer type-specific functions (9), and our data revealed that MC2 MCs and MC4 MCs correspond to these two populations (**Figure 4D**).

Using quantitative analysis, we observed that the frequency of TNF^+^ MCs was greater than that of VEGFA^+^ MCs in all tissues of ccRCC patients (**Figure 3D**). TNF^+^ MCs release chemokines to recruit T cells and secrete TNF-α to further regulate T-cell activity. MCs also function as local antigen-presenting cells for T cells by upregulating MHC-II and costimulatory molecules. All of these effects may result in overall MCs eventually exerting predominantly antitumor effects on ccRCC (**Figure 3C**; **Supplementary Figure S1C**). This hypothesis is consistent with that of the TCGA database, indicating the critical role of MCs in the prognosis of ccRCC patients and their potential as protective prognostic biomarkers (**Figure 4C**). However, in terms of the ratio of TNF^+^ to VEGFA^+^ MCs, the ratios of tissues at the tumor core (1.8) and tumor rim (1.2) were significantly lower than those of adjacent normal tissues (16) (**Figure 4E**). These findings suggest that in ccRCC tissues, the proangiogenic effect is enhanced due to the increased quantity and function of MC4, especially at the tumor-normal interface (the leading edge of the tumor) (**Figures 3D-H**). These findings prompted our curiosity about the production and function of MC4 at the tumor-normal interface of ccRCC for further exploration.

In conclusion, the reduction in the number of proliferating and resting MCs and the shift from MC0 MCs to MC4 MCs in activated MCs in ccRCC tissues contribute to a net balance of antitumor and protumor signals, further determining the antitumor role of MCs in ccRCC.

## MC4 is linked to promoted tumor angiogenesis and decreased survival via interactions with IL1B^+^ macrophages

**Figure 5:**
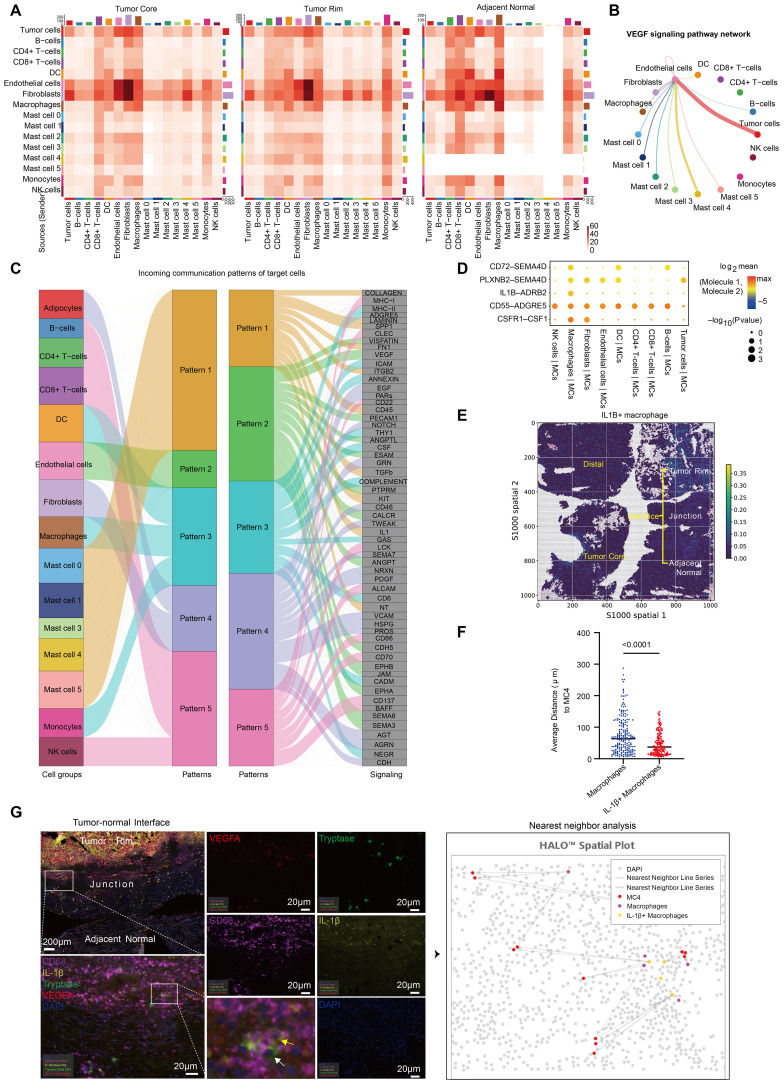
MC4 is linked to promoted tumor angiogenesis and decreased survival via interactions with IL1B+ macrophages. **(A)** Heatmap of the number of significant ligand‒receptor interactions between major cell types and each MC population. **(B)** Network of the VEGF signaling pathway between major cell types and each MC population. The thickness of the line represents the strength of the intercellular signaling pathway. **(C)** River map of the incoming communication patterns of the target cells. **(D)** CellPhoneDB analysis of ligand‒receptor interactions between MCs and macrophages. The size and color of the dots indicate the strength of the interaction between different cell groups. **(E)** Spatial plot showing the spatial distribution of IL1B+ macrophages. **(F)** Quantification of the proximity of MC4 cells to the nearest IL-1β+ macrophages and other macrophages. Each point in the plot represents the average of five similar values. **(G)** Tissues from the tumor-normal interface of 15 ccRCC patients were stained and analyzed by multiplex immunofluorescence (mIHC) for Tryptase, VEGFA, CD68 and IL-1β. The white arrows point to cells that are positive for VEGFA and tryptase (MC4), and the yellow arrows point to cells that are positive for CD68 and IL-1β (IL-1β+ macrophages). Strategy for analyzing the proximity of MC4 cells to the nearest IL-1β+ macrophages and other macrophages (right).

To further explore the underlying reasons for MC4 enrichment in ccRCC and determine its functions, we calculated the potential ligand−receptor pairs in each ccRCC tissue via CellChat (26). We found that the number of MC2 interactions with other cell types was greatest in adjacent normal tissues, whereas the number of MC2 and MC4 interactions with other cell types was even greater in ccRCC tissues (tumor core and rim) (**Figure 5A**; **Supplementary Table S9**). More importantly, the VEGF signaling pathway was significantly enriched in tumor tissues compared with adjacent normal tissues and was strongly associated with MC4, further corroborating the role of MC4 in promoting tumor angiogenesis in ccRCC (**Figure 5B**).

Next, to explore whether active cell−cell interactions regulate the effects of MC4, we used CellChat and discovered the strongest interactions between MC4 and macrophages, endothelial cells, and fibroblasts (**Figure 5A**). In addition, we predicted the ligands in the tumor rim that may drive the MC4 phenotype in ccRCC and noted interleukin (IL)-1 (**Figure 5C**). Because macrophages presented the highest level of IL1B expression among all cells (**Supplementary Figure S4A**), we used CellPhoneDB (27) to characterize the interactions between macrophages and MCs in ccRCC and found that IL1B-ADRB2, CSF1R-CSF1, CD55-ADGRE5, PLXNB2-SEMA4D, and CD72-SEMA4D interactions link MCs to macrophages. Among them, IL1B-ADRB2 is a ligand−receptor pair that is significantly and specifically enriched between macrophages and MCs (**Figure 5D**). ADRB2 has been reported to be associated with triggering angiogenesis, which induces and maintains tumor vascularization to support expansive tumor growth (28). Moreover, we further analyzed the expression of ADRB2 in MCs and found that it was specifically and most highly expressed in MC4 (**Supplementary Figure S4B**).

Furthermore, spatial transcriptomics data revealed that IL1B^+^ macrophages also clustered at the tumor-normal interface in ccRCC (**Figure 5E**), which is consistent with previous reports (11). Immune cells rely on transient physical interactions with other populations to regulate their function (29). We stained tissue sections of the tumor-normal interface from 15 ccRCC patients for CD68, IL-1b, VEGFA and tryptase and analyzed the proximity of each MC4 cell to the nearest IL-1β^+^ macrophage and other macrophages (**Figure 5F**; **Table 1**). The results revealed that cells in MC4 were closer to IL-1β^+^ macrophages than to other macrophages (**Figures 5F; G**), which further supported the existence of spatial colocalization of IL-1β^+^ macrophages with MC4, further supporting a potentially critical role for IL1β^+^ macrophages-MCs interactions at the interface of ccRCC (29–31): the presence of IL1β^+^ macrophages may drive the onset of MC heterogeneity in ccRCC, namely, the generation of MC4, which plays a protumor angiogenic role.

In summary, MC4 cells constitute a group of MC cells with the most activated phenotype in ccRCC, exhibiting the highest expression of protease genes as well as other MC-related receptor and mediator genes. High expression of VEGFA promotes tumor angiogenesis, which supports cancer development. During the progression of ccRCC, the production of IL-1β by IL-1β^+^ macrophages may have contributed to the formation of MC4. Therefore, targeting IL-1β in ccRCC may achieve antitumor effects by curbing the production of MC4, which is more beneficial for patient survival.

## Discussion

Our study investigated the heterogeneity and role of MCs in ccRCC via single-cell transcriptomic sequencing. Importantly, MCs in ccRCC are characterized by an antitumor preference, which is consistent with their association with a better prognosis. Another significant finding was the observation of MC4 in ccRCC. MC4 is a cluster of MCs with the highest expression of MC-related receptor and mediator genes and significantly expresses VEGFA. Further investigation indicated that the upregulation of IL1B expression in macrophages in ccRCC may play a critical role in the production of MC4, ultimately exerting a protumor angiogenic role in ccRCC patients. Our findings revealed that ccRCC has the potential to respond to MC-targeted immunotherapy, but the underlying mechanisms and possibilities need to be further explored.

In humans, MCs are classically defined by their protease content, divided into subpopulations coexpressing tryptase and chymase (MCTC), localized in the skin, mucosal subepithelium, and peripheral connective tissues, or subpopulations expressing tryptase (MCT) in the absence of chymase, predominantly in the mucosal epithelium (32). Immunohistochemical staining studies also revealed differences in the expression of cytokines between different MC subsets. MC expansion in tumors was initially described in the 19th century, and determining the roles of MCs in cancers has been challenging (33).

Recently, the widespread use of technologies such as single-cell sequencing has deepened the understanding of MC heterogeneity. According to their different roles in tumors, MCs have been classified into numerous subtypes, including anti-tumorigenic MCs and pro-tumorigenic MCs (9; 34). In our study, we defined activated MCs (MC0 and MC4), resting MCs (MC3), and proliferative MCs (MC2) in ccRCC on the basis of the high or low expression of MC receptors and mediator genes as well as the high expression of proliferation-related genes, respectively. We observed that the proportion of activated MCs in ccRCC tissues was generally consistent with that in adjacent normal tissues, but there was a shift in the activated subpopulation (MC0 to MC4), while the proportions of proliferating MCs and resting MCs were lower. These findings provide a new perspective for revealing the function of MCs in ccRCC from the perspective of MC heterogeneity.

In addition, MC1 and MC5 were classified as other MCs. We carried out KEGG pathway analysis on MC1 and found that it was highly enriched for oxidative phosphorylation metabolic pathways (**Supplementary Figure S5A**), which suggests that this group of cells may be related to active energy metabolism, enhanced cell proliferation and differentiation, and the modulation of signal transduction (35). MC5 is notably characterized by high expression of FER (ferritin) (**Supplementary Figure S1B**). Previous studies have shown that MCs that highly express FER in tumors influence the disease process by regulating iron metabolism and inflammatory responses. Studies on FER-deficient mice have shown that it plays a prominent role in regulating mast cell activation (36). FER promotes activated mast cell chemotaxis by activating p38 kinase (37). In this case, MC1 and MC5 tend to exhibit a relatively activated state. However, some previous studies reported that damaged and dead cells often exhibit extensive mitochondrial contamination (38; 39), which is consistent with the fact that more necrosis occurs in tumor tissues than in normal tissues. Therefore, these cells were not classified as activated MCs and were excluded from further analyses. However, their biological function needs to be further explored.

In our study, we used an MC signature to assess MC density in tumor and normal tissues from bulk RNA-seq data. Our results revealed that MC density was significantly lower in most tumors, with the significantly highest MC density in ccRCC. This finding suggests that increased MC density plays a protective role during ccRCC progression.

The role of MCs in cancers remains controversial (25; 40–42). A recent single-cell study of MCs across cancers divided tumor MCs into two subtypes: protumorigenic MCs, characterized by a low TNF^+^/VEGFA^+^ ratio, and antitumorigenic MCs, characterized by a high TNF^+^/VEGFA^+^ ratio (9). The role of the innate immune system in determining the clinical outcome of patients with ccRCC remains ambiguous. In our study, MC2 and MC4 represented TNF^+^ and VEGFA^+^ MCs, respectively. The TNF^+^/VEGFA^+^ ratio was high in adjacent normal tissues (ratio=4), which was lower than that in tumor tissues, with the lowest ratio occurring at the tumor rim (ratio=1.2). These findings suggest that overall, MCs mainly exert antitumor effects on ccRCC, which is consistent with the findings of several previous reports. Moreover, in the TCGA cohort, patients with abundant MC infiltration had longer overall survival (P < 0.001), validating our findings. However, renal tumors did not show antitumor effects according to the pancancer analysis of Cheng et al (9). Instead, they exhibited protumorigenic effects because of the low TNF^+^/VEGFA^+^ ratio, in contrast to our findings. In our view, this can be explained. Our data revealed that MC4 was increased in tumor tissues, especially at the tumor–normal interface, compared with adjacent normal tissues. Our results suggest that the production of MC4 is due to the presence of many ILB^+^ macrophages at the tumor-normal interface, which secrete IL-1β that acts on ADRB2 on VEGFA^+^ MCs. They cause MC4 to be produced in large numbers and play a role in promoting tumor angiogenesis. Since all four patients in our study were at stage 1 (**Supplementary Table S1**), the number of ILB^+^ macrophages in their TME was low, which may have caused the number of induced VEGFA^+^ MCs to be lower than that of TNF^+^ MCs. In addition, as far as the TNF^+^/VEGFA^+^ ratio in ccRCC is concerned, both our study and Cheng et al.’s study were close to 1; however, in contrast, more large-sample studies are needed in the future to further clarify the function of MCs in ccRCC, or the discussion needs to be further refined according to the tumor stages.

IL1B is a cytokine that is a central mediator of cell−cell interactions in the inflammatory TME. ADRB2 has been reported to be associated with triggering angiogenesis, which induces and maintains tumor vascularization, thereby supporting expansive tumor growth. IL1B plays a key role by binding to the receptor ADRB2 on MCs. Our cellular analysis of ccRCC samples revealed that IL1B was enriched in macrophages. In addition, we found that ADRB2 was significantly expressed in MC4 in ccRCC samples. These findings suggest that IL1B expression in macrophages may be a key upstream factor for MC4 production in ccRCC. These results offer new insights into the molecular mechanisms underlying MC functional heterogeneity in ccRCC and emphasize the potential of the IL1B/ADRB2 axis as a therapeutic target for cancer. For example, blocking IL1B expression in IL1B^+^ macrophages could prevent the production of angiogenesis-promoting MC4 via the IL1B/ADRB2 pathway, resulting in the inhibition of tumor growth, a reduction in the TNF^+^/VEGFA^+^ ratio, and an increase in patient prognosis. Previously, researchers have studied the ability of IL-1β expression to mediate RCC cell invasion through the von Hippel−Lindau (VHL) null cell line model (43). Several studies have demonstrated the role of blocking IL-1β in promoting tumor regression in renal cancer (44, 45), and our study provides new ideas about the possible mechanisms of this therapeutic approach.

Furthermore, we used spatial transcriptome analysis to validate the spatial distribution of the various populations of MCs, where MC4 clustered at the tumor-normal interface and colocalized with IL1B^+^ macrophages. These findings provide new insights into the complexity and criticality of the TME at the tumor–normal interface.

To the best of our knowledge, the strength of this study is that we performed the first study, which focused mostly on MC populations in ccRCC patients. In addition, this study was verified *in vitro* via mIHC on tissue sections from 15 ccRCC patients, which can provide insights for other researchers in the future. However, there are still several limitations of this study. In the future, these findings need to be verified in a wider population, and the feasibility and mechanism of MC-targeted immunotherapy for the treatment of ccRCC remain to be further explored.

In conclusion, our study demonstrated increased MC density and comparable MC activation in ccRCC, which are specific compared with those in normal tissues and other cancer types. However, we found that the activation phenotype shifted from a CTSG-high MC0 phenotype to a more activated VEGFA^+^ MC phenotype, and the proliferative and resting phenotypes were reduced. Overall, our results revealed that MCs have a TNF^+^/VEGFA^+^ ratio of >1 and thus play an antitumor role in ccRCC. However, there may be cellular interactions and an IL1B-ADRB2 axis to massively expand MC4 at the tumor-normal interface in the TME, exerting a proangiogenic role and potentially promoting tumor progression. By deciphering the heterogeneity of MCs in ccRCC at the single-cell level, our study may contribute to the development of effective immunotherapies targeting MCs in ccRCC.

## Methods

### Materials

This study utilized several public datasets, including four scRNA-seq datasets (EGAD00001008030, GSE210038, GSE202374, and GSE207493) and five bulk RNA sequencing (bulk RNA-seq) datasets. The bulk RNA-seq datasets include high-throughput sequencing data from TCGA-KIRC and four Gene Expression Omnibus (GEO) microarray datasets (GSE40435, GSE36895, GSE66271, and GSE66270). Transcriptomic and clinical information from the GEO datasets was obtained from the GEO database (https://www.ncbi.nlm.nih.gov/geo/).

### Human subjects

The collection of human tumor and kidney tissues for the study was approved by the Ethics Committee of Xijing Hospital (no. XJYY-LL-FJ-059). Informed consent was acquired from all the donors.

### Single-cell RNA sequencing

Kidney tumors were removed on the day of surgery and placed on ice. The samples consisted of 3 different areas from the tumor core, tumor-normal interface, and adjacent normal tissue. Tissues were cut and digested into single-cell suspensions. CD45^+^ cells were subsequently isolated via the Human CD45 Positive Selection Kit (100-0107, StemCell) according to the manufacturer’s protocol. CD45^+^ cells were mixed 9 to 1 with CD45^-^ cells as sequencing samples. After performing quality control, single-cell libraries were constructed for scRNA-seq following the manufacturer’s instructions. Sequencing was performed via the second-generation sequencing platform.

### Single-cell sequencing data processing (publicly available datasets)

Three ccRCC-related scRNA-seq datasets (GSE202374, GSE2027493, and GSE210038) were downloaded from the GEO database. The “Seurat” package was employed to load and analyze the datasets, and the “harmony” package was used to remove batch effects. The canonical marker genes were used to define the mast cell population, and then, the mast cells from each dataset were extracted and integrated into one Seurat object. The “Find All Markers” function was employed to detect DEGs among each cell group.

### Cell communication analysis

To study the interactions between MCs and major cell types, we used the methods of CellChat (26) and CellPhoneDB (27) to identify putative ligands and receptors on the basis of their expression on each cell. The CellChat package (version 1.6.1) was used to investigate the ligand−receptor pairs between disparate cell types, as previously documented. The ligands and receptors expressed in more than 10% of the cells within the specific cluster were subsequently selected for further analysis. The interactions among distinct cell subpopulations through putative ligand−receptor pairs were illustrated via the ggplot2 package. The CellPhoneDB (version 2.0.6) utilized the cluster annotation and raw counts derived from our scRNA-seq data to compute cell−cell communication between distinct cell types. In this process, the default ligand−receptor pair information was utilized, with only receptors and ligands with expression in more than 15% of the cell subtypes considered; the P values were calculated via a 1000-time permutation test, with values greater than 0.05 indicating significant enrichment among the interacting ligand−receptor pairs in all interacting pairs of two cell types. To facilitate a comprehensive and systematic analysis of cell−cell communication, we undertook a reclustering of the distinct cell types.

### RNA velocity analysis

The RNA velocity analysis was conducted via the scvelo R package (version 0.3.2) (24), which was employed to recount unspliced and spliced reads from prealigned BAM files of single-cell RNA sequencing data. Subsequently, RNA velocity values were calculated for each gene in each cell, with the resulting RNA velocity vectors then embedded into a low-dimensional space utilizing the scvelo pipeline. Thereafter, the developmental trajectories were derived by embedding the RNA velocity vectors within the UMAP space.

### Functional and pathway enrichment analysis

GO enrichment analysis was performed between tumor and normal MCs via the hallmark gene set.

### Prognosis analysis of MC signature genes and MC signature genes

To evaluate the role of the MC signature in the prognosis of each cancer, we used Kaplan−Meier models and univariate Cox regression. We analyzed five types of prognostic data, including OS, DSS, DFS, DFI, and PFI. In addition, we performed disease-free survival and overall survival analyses of MC signature genes in the TCGA-KIRC cohort via GEPIA2.

### Spatial transcriptomics analysis

Freshly frozen samples from the tumor core, tumor-normal interface and distal kidney tissues were embedded in optimal cutting temperature medium (OCT) and sectioned according to the manufacturer’s protocol. The generated sections were selected on the basis of HE staining, with a focus on morphology. After tissue optimization, library preparation and sequencing were performed according to the manufacturer’s protocol.

### Multiplex immunohistochemistry

The sections, 4 μm thick, were placed in an oven maintained at 60°C overnight. The slides were deparaffinized in xylene and subsequently dehydrated in a graded series of alcohol at concentrations of 100%, 95%, 90%, 85%, and 75%. Following the blocking of endogenous peroxidase activity, mIHC was performed via the Manual Opal 7-Color IHC Kit (Phenoptics, NEL811001KT) according to the manufacturer’s protocol. Scanning images were obtained via a PhenoImager HT scanner (Akoya Biosciences), and image analysis was carried out via HALO software. The staining panels were as follows:

### Statistical analysis

scRNA-seq and spatial transcriptome sequencing were carried out by Biomarker Technologies Co., Ltd. (Qingdao, China). Basic analysis of the scRNA-seq data was performed via BMKCloud (www.biocloud.net). For survival analysis, we used the log-rank and univariate Cox methods. All mIHC images were analyzed with HALO software. Statistical analyses were performed with GraphPad Prism software, version 10.1.2. Significant differences in the tests were analyzed via unpaired t tests or one-way ANOVA tests with a two-sided distribution. For all primary hypothesis tests, we also report P values of multiple comparisons based on Benjamini−Hochberg adjustments; this takes into account the possibility of false positive results, which increases with the number of hypotheses tested. In addition, we used a chi-square test for independence to assess whether these clinical features had an impact on the results of the analysis, with the data organized into a 2x2 contingency table. The analysis was conducted via SPSS. A p value less than 0.05 was considered statistically significant.

## Data Availability

The data presented in the study are deposited in the Sequence Read Archive (SRA) repository, accession number PRJNA1202509.

## Supplementary material

Supplementary Figure S1 The batch effect correction in our study, related to **Figure 1**. **(A)** Distribution of cells in clusters colored according to tissue types. **(B)** Representation of cells from different tissues within each cluster based on normalized cell numbers per tissue, demonstrating that all clusters contain cells from multiple patients and are therefore not related to batch effects. **(C)** Frequency of clustered cells shown as a fraction of total cells for each tissue.

Supplementary Figure S2 Marker gene expression for MCs in the three cohorts, related to **Figure 1**. UMAP plots showing marker gene expression for MCs in the 3 cohorts (GSE210038, GSE202374, and GSE207493) dataset.

Supplementary Figure S3 DEGs of MC subsets in ccRCC, related to **Figure 3**. **(A)** Heatmap showing the expression of cell proliferation-related genes in MC clusters. **(B)** Heatmap showing the marker genes of each MC cluster. All the marker genes are listed in **Supplementary Table S2**. **(C)** Volcano plots showing the DEGs between MC2 (left) and MC3 (right). **(D)** UMAP plot of MCs colored by cluster in the three cohorts. **(E)** Dot plot showing the expression of MC-related receptor and mediator genes across different MC clusters in the three cohorts. **(F)** A schematic diagram showing the correspondence between the samples of scRNA-seq and spatial transcriptomes. **(G)** Representative images of the global views of the tissues from the distal normal kidney, tumor core and tumor-normal interface.

Supplementary Figure S4 Genes related to cellular interactions are shown in **Figure 5**. **(A)** UMAP plot showing IL1B expression in all cell compartments. **(B)** Violin plot showing ADRB2 expression in MC clusters.

Supplementary Figure S5 Discussion of the cluster of “other MCs”. **(A)** Enriched pathways in MC1 according to the KEGG pathway enrichment analysis.
