# Supplementary figures and images for: Single-cell transcriptomics reveals the heterogeneity and function of mast cells in human ccRCC
Source: Front Immunol. 2025 Jan 7;15:1494025. doi: 10.3389/fimmu.2024.1494025 (PMC11747552; doi:10.3389/fimmu.2024.1494025)

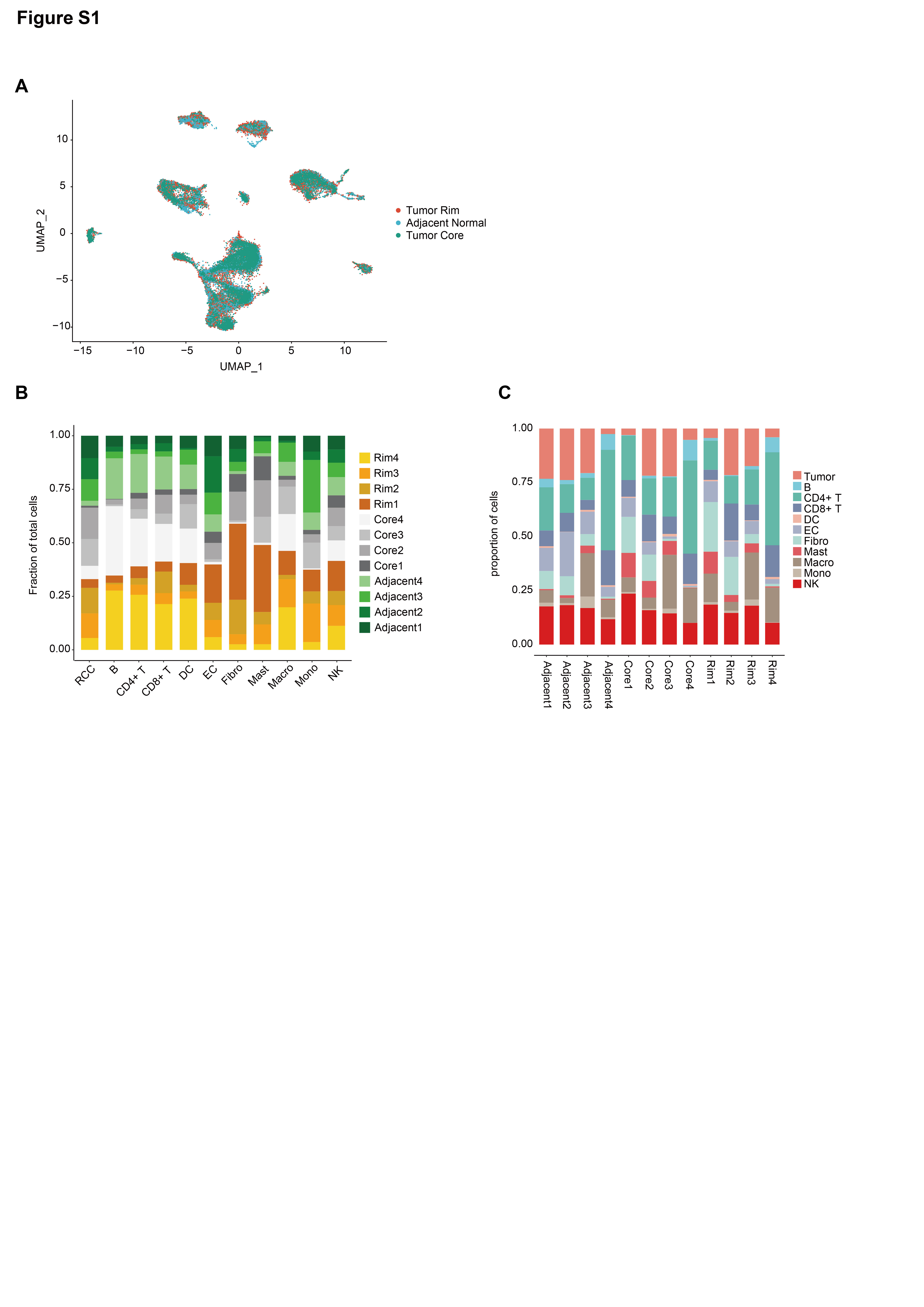

Supplement: Supplementary Figure S1 — The batch effect correction in our study, related to Figure 1 . (A) Distribution of cells in clusters colored according to tissue types. (B) Representation of cells from different tissues within each cluster based on normalized cell numbers per tissue, demonstrating that all clusters contain cells from multiple patients and are therefore not related to batch effects. (C) Frequency of clustered cells shown as a fraction of total cells for each tissue. [file Image1.jpeg]

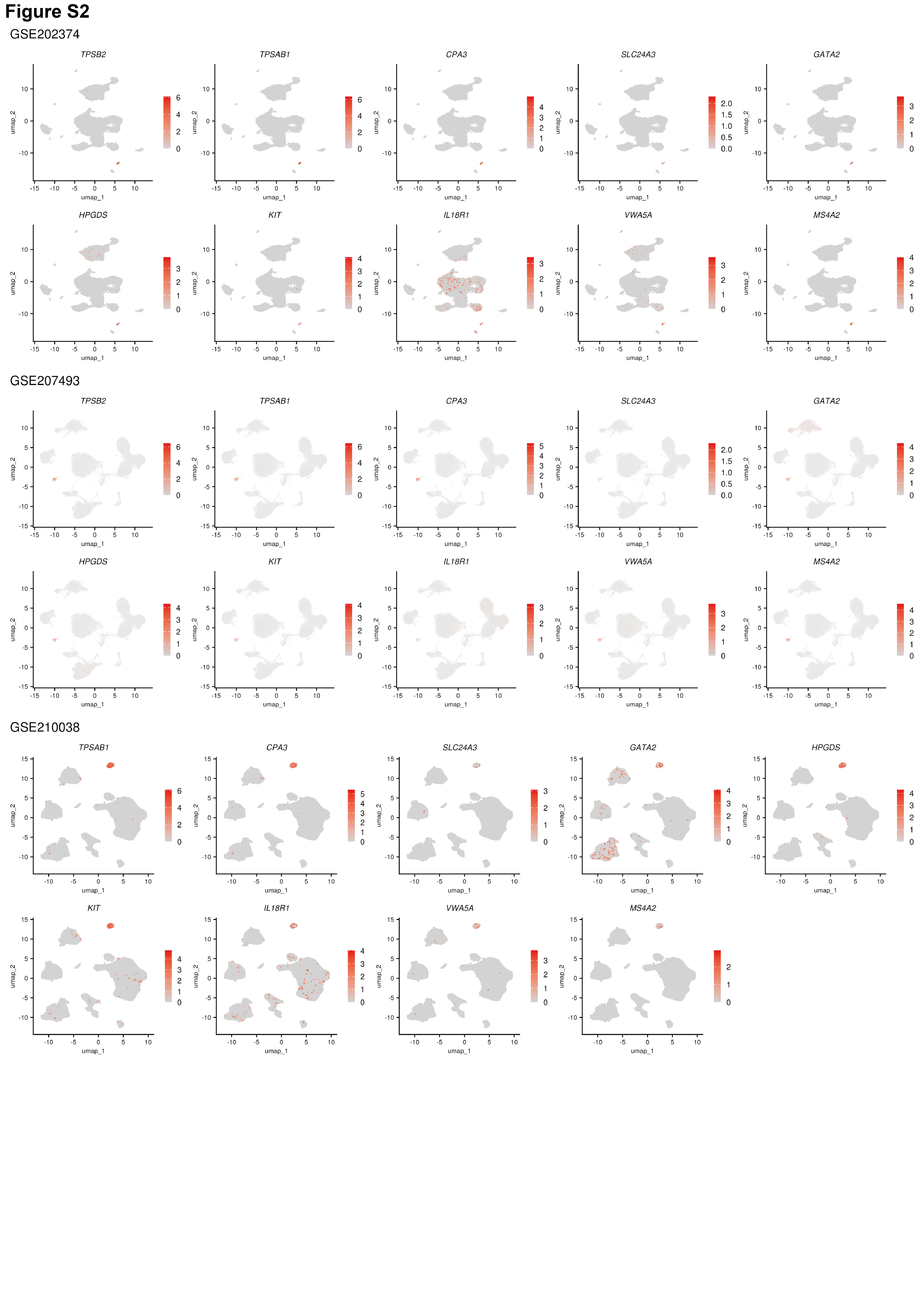

Supplement: Supplementary Figure S2 — Marker gene expression for MCs in the three cohorts, related to Figure 1 . UMAP plots showing marker gene expression for MCs in the 3 cohorts (GSE210038, GSE202374, and GSE207493) dataset. [file Image2.jpeg]

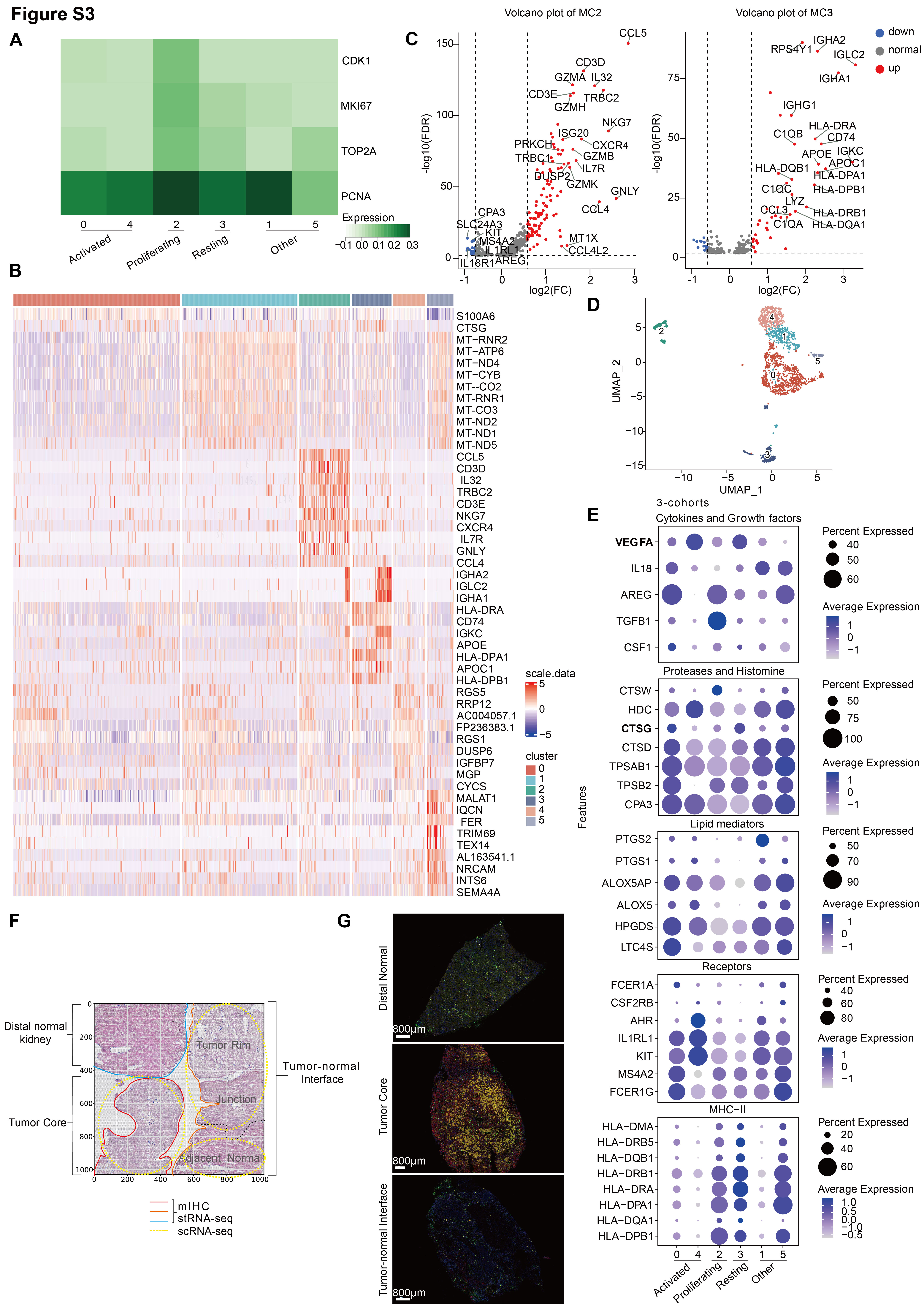

Supplement: Supplementary Figure S3 — DEGs of MC subsets in ccRCC, related to Figure 3 . (A) Heatmap showing the expression of cell proliferation-related genes in MC clusters. (B) Heatmap showing the marker genes of each MC cluster. All the marker genes are listed in Supplementary Table S2 . (C) Volcano plots showing the DEGs between MC2 (left) and MC3 (right). (D) UMAP plot of MCs colored by cluster in the three cohorts. (E) Dot plot showing the expression of MC-related receptor and mediator genes across different MC clusters in the three cohorts. (F) A schematic diagram showing the correspondence between the samples of scRNA-seq and spatial transcriptomes. (G) Representative images of the global views of the tissues from the distal normal kidney, tumor core and tumor-normal interface. [file Image3.jpeg]

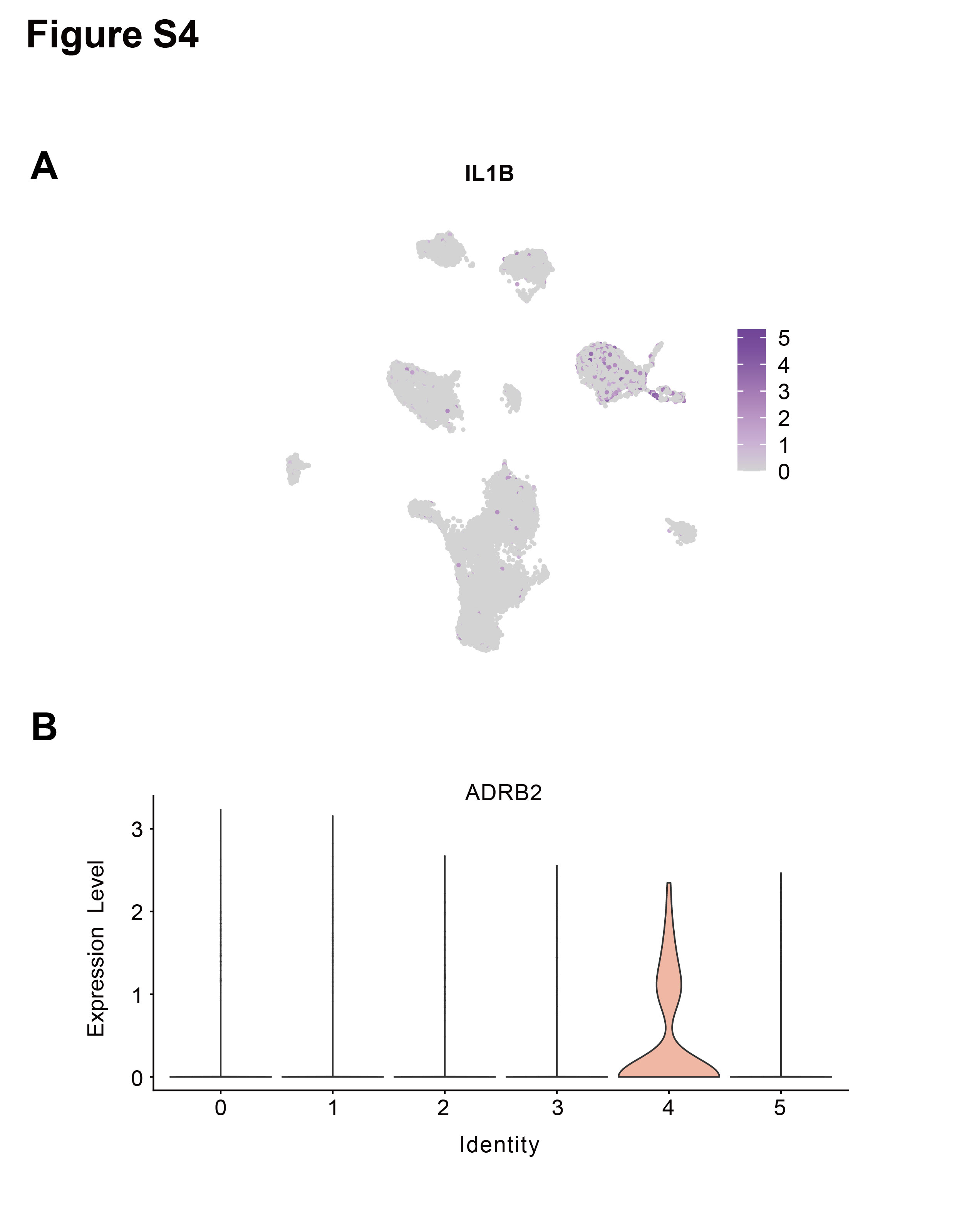

Supplement: Supplementary Figure S4 — Genes related to cellular interactions are shown in Figure 5 . (A) UMAP plot showing IL1B expression in all cell compartments. (B) Violin plot showing ADRB2 expression in MC clusters. [file Image4.jpeg]

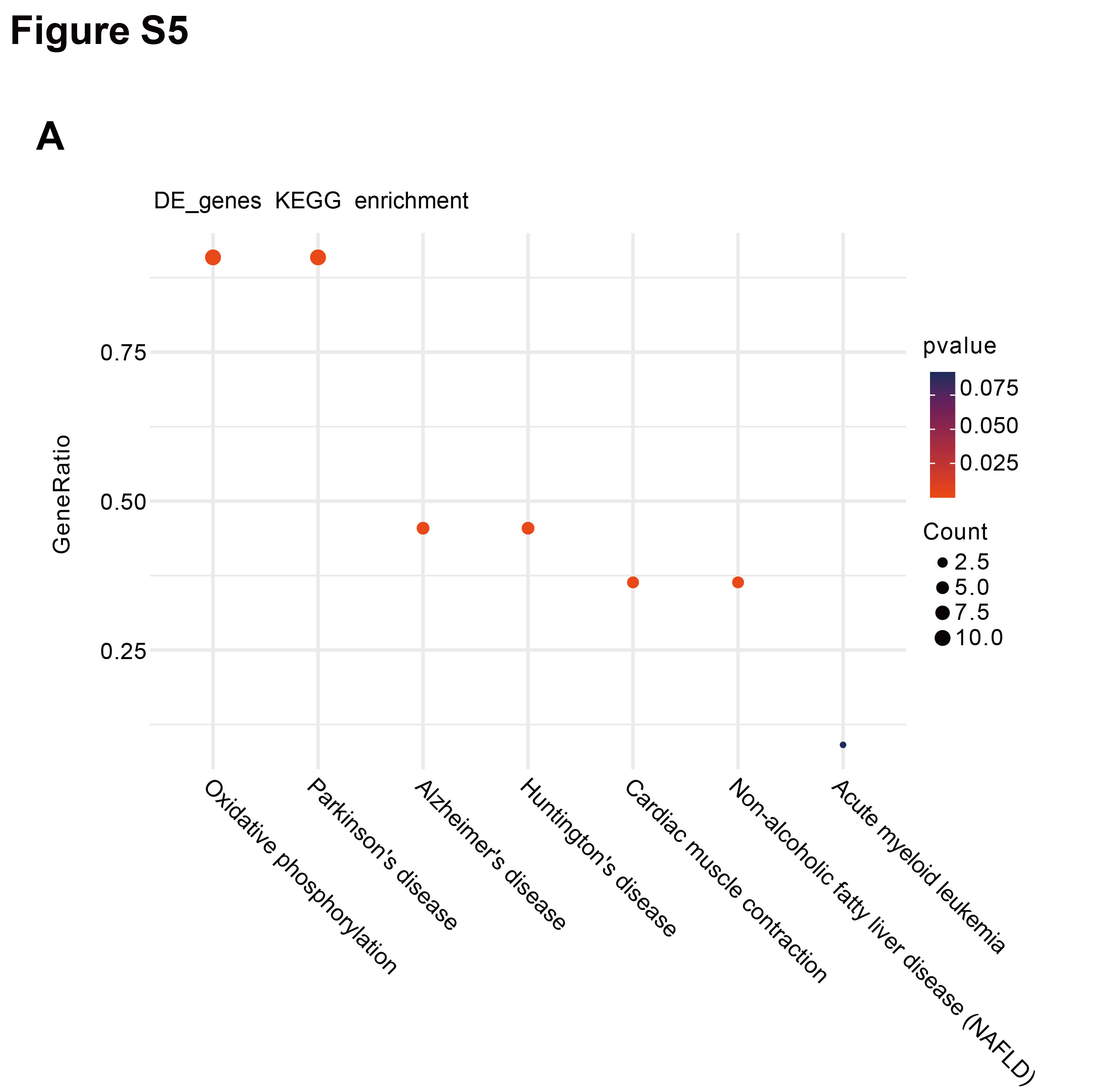

Supplement: Supplementary Figure S5 — Discussion of the cluster of “other MCs”. (A) Enriched pathways in MC1 according to the KEGG pathway enrichment analysis. [file Image5.jpeg]
